# Comparative Gut Proteome of *Nyssomyia umbratilis* from Leishmaniasis Endemic and Non-Endemic Areas of Amazon Reveals Differences in Microbiota and Proteins Related to Immunity and Gut Function

**DOI:** 10.3390/microorganisms13061304

**Published:** 2025-06-04

**Authors:** Antonio Jorge Tempone, Guilherme Ian Spelta, Victor Ramos de Almeida, Daniel Machado Giglioti, Erika Moutinho Costa, Izabela Mathias, Helena Vargas, Thais Lemos-Silva, Ana Carolina Pedro dos Santos Ribeiro, Eric Fabrício Marialva, Cláudia Rios-Velasquez, Michel Batista, Marlon Dias Mariano dos Santos, Felipe Arley Costa Pessoa, Yara Maria Traub-Csekö

**Affiliations:** 1 Laboratório de Biologia Molecular de Parasitas e Vetores, Instituto Oswaldo Cruz, Fiocruz, Rio de Janeiro 21040-900, RJ, Brazil; 2 Laborátório de Ecologia e Doenças Transmissíveis na Amazônia—ILMD Fiocruz Amazônia, Manaus 69057-070, AM, Brazil; 3 Plataforma de Espectrometria de Massas-RPT02H, Curitiba 81350010, PR, Brazil; 4 Laboratory for Structural and Computational Proteomics, Carlos Chagas Institute, Curitiba 81350-010, PR, Brazil; 5 Analytical Biochemistry and Proteomics Unit, Instituto de Investigaciones Biológicas Clemente Estable, Institut Pasteur de Montevideo, Mataojo 2020, Montevideo 11400, Uruguay

**Keywords:** Leishmaniais, *Nysomyia umbratilis*, *Leishmania guyanensis*, gut proteomics, microbiota, sand fly and leishmania interaction

## Abstract

The northern region of Brazil is endemic for American Tegumentary Leishmaniasis (ATL) primarily caused by *Leishmania guyanensis* and transmitted by the sand fly *Nyssomyia umbratilis*. The disease occurs at different rates in the municipalities of Manacapuru (MAN) and Rio Preto da Eva (RPE), located in the state of Amazonas. Despite their geographic proximity and separation by the Rio Negro, MAN has a low incidence, whereas RPE reports a significantly higher number of cases. Since the vector is present in both locations, potential biological differences in *N. umbratilis* may influence transmission. Previous studies suggested genotypic and phenotypic differences in *N. umbratilis* from both localities. To investigate the molecular factors underlying their potentially differential vectorial capacities, we performed a comparative proteomic analysis of dissected insect intestines from both localities. Our results revealed that sand flies from MAN showed a higher abundance of proteins related to gene transcription, protein translation, amino acid and proton transport, innate immune response and intestinal motility. Since the importance of microbiota has previously been shown in parasite–vector interactions, we also identified bacteria from both vector populations. We detected bacteria specific to each population and, exclusively in MAN, some species described in the literature as having parasiticidal properties. These findings highlight molecular and microbial peculiarities that could contribute to the observed difference in ATL prevalence in the two areas.

## 1. Introduction

*Nyssomyia umbratilis* (Ward & Fraiha) is considered the primary vector of *Leishmania guyanensis*, the principal etiological agent of American Tegumentary Leishmaniasis (ATL) in the north of the South American Hemisphere, in countries such as French Guiana, Suriname and the Brazilian Amazon rainforest [1]. ATL has a zoonotic nature and is considered a polymorphic disease that can affect the skin or mucous membranes. *L. guyanensis* causes skin lesions in the form of single ulcers and can undergo lymphatic metastases, causing lymphangitis and lymphadenopathy [2].

Although ATL is prevalent in the state of Amazonas (AM), its distribution differs between Manacapuru (MAN) and Rio Preto da Eva (RPE), situated in the metropolitan area of Manaus-AM. These locations are approximately 180 km apart and are separated by the Negro river. While RPE is considered endemic for ATL, MAN presents no significant number of ATL cases [3], despite the presence of the *N. umbratilis* vector in both locations [4]. Data from the Brazilian Ministry of Health revealed that MAN had approximately 10 times fewer cases of leishmaniasis than RPE per 100,000 inhabitants. From 2018 to 2022, RPE had an annual average of 178 cases, while MAN had an average of 16 cases Ministerio da Saude. Available online: https://www.gov.br/saude/pt-br/assuntos/saude-de-a-a-z/l/lt/situacao-epidemiologica (accessed on 12 September 2024).

The hypothesis of allopatric speciation among the vectors has been proposed to explain this potentially different vectorial capacity phenomenon [5]. A more recent study investigating Cytochrome Oxidase I (COI) genetic diversity among *N. umbratilis* populations collected from nine locations on the banks of the Amazon and Negro river in central Amazonia corroborated previously observed distinctions between sand flies from MAN and RPE [6]. Furthermore, ex vivo studies revealed particularities in the ability of the parasites to adhere to the vector’s gut [7], critical for successful infection, with the parasites failing to attach to MAN vectors’ guts, while they were able to adhere to RPE insects’ guts.

The sand fly gut plays a fundamental role in the processes related to the infection, development and transmission of the etiological agent of ATL and all leishmaniasis. One important step for the success of vector infection by the parasite is the attachment to the intestinal wall at the end of the digestive process and the degradation of the peritrophic matrix. At this point, the pathogen is released into the gut, and the attachment to the gut helps to avoid elimination along with vector feces. Another fundamental step involves the differentiation of the parasites into infective metacyclic forms. All of these take place inside the phlebotomine’s digestive tract, where the parasites interact with intestinal proteins and the resident microbiota [8].

To investigate potentially relevant molecular differences between the *N. umbratilis* populations from MAN and RPE and considering that this sand fly species cannot be colonized and that parasite development within the vector occurs entirely inside its intestinal tract, we conducted a proteomic study of dissected guts from sand flies collected in both regions. This analysis also enabled the identification of bacterial peptides from the vector microbiota.

Our results demonstrate that, although the two populations share similar gut protein composition, they can be separated into two distinct groups based on expressed proteins. Each group presented a different set of differentially detected proteins that showed significant interaction rates, related to gene transcription and translation. In MAN, we identified a higher abundance of proteins associated with motility and the transport of protons and amino acids, whose higher activity could be associated with greater peristalsis and intestinal pH in these insects. The transcriptional profiles of genes coding for these differentially and exclusively detected proteins were evaluated, confirming the mass spectrometry proteomic data.

Metaproteomic analysis showed that the most abundant bacterial genera are common to the microbiome of both populations; however, some genera are uniquely present in each population. In MAN, we found two bacterial species, *Raoultella terrigena* and *Shewanella* sp., which may contribute to the observed vectorial difference between the populations of *N*. *umbratilis* from MAN and RPE, since both show parasiticidal properties [9,10].

Our findings revealed molecular and microbial differences that might help explain the observed disparities in leishmaniasis endemicity in the two regions.

## 2. Material and Methods

### 2.1. Sand Flies

*N. umbratilis* were collected from the municipalities of MAN and RPE, identified, dissected and separated into carcass and intestine. 

### 2.2. Mass Spectrometry Analysis

Three pools of 20 insect intestines from MAN and RPE were macerated in 6 M urea, 2 M thiourea and 10 mM 4-(2-hydroxyethyl)-1-piperazineethanesulfonic acid (HEPES). The extracts were subjected to SDS-PAGE followed by in-gel digestion. Briefly, proteins were reduced with 10 mM Dithiothreitol (DTT) and 50 mM ammonium bicarbonate (ABC) and alkylated with 55 mM iodoacetamide 50 mM ABC. Then, the samples were digested in 50 mM ABC with 12.5 ng/µL trypsin (Promega, cat. V5113, Madison, WI, USA) at 37 °C for 18 h. Peptides were then extracted from the gel twice with 30% MeCN, 3% TFA, twice with MeCN, concentrated in a speed vac and desalted with homemade C18 spin columns. The peptides were analyzed in triplicate by liquid chromatography tandem–mass spectrometry (LC-MS/MS) in a Thermo Scientific Ultimate 3000 RSLCnano coupled to an Orbitrap Fusion Lumos (mass spectrometry facility RPT02H/Carlos Chagas Institute–Fiocruz, Curitiba, PR, Brazil). Peptide separation was carried out in 15 cm (75 µm inner diameter) fused silica, in-house packed with reversed-phase ReproSil-Pur C18-AQ 3 µm resin (Dr. Maisch GmbH, Ammerbuch-Entringen). Chromatography runs used phase A composed of 0.1% formic acid and phase B 80% MeCN, 0.1% formic acid in a flow rate of 250 nL/min. The gradient was from 6.2% to 33% B in 120 min and from 33% to 50% B in 10 min. A voltage of 2.3 kV was applied in a pneu-Nimbus nano source (PST) for peptide ionization. The mass spectrometer operated in a data-dependent acquisition mode. Survey full-scan MS spectra (at a 300–1500 *m*/*z* range) were acquired in the Orbitrap analyzer with a resolution of 120,000 at *m*/*z* 200. The most intense ions were sequentially isolated, fragmented and analyzed in the orbitrap at 15,000 resolution in a 2s cycle. The internal calibration option was enabled in all full scans to improve the mass accuracy of precursor ions.

### 2.3. Protein Identification and Quantitation

Protein identification was performed with the MaxQuant algorithm [11,12] version 1.4.1.2. The default parameters of the software were used for all analysis steps, unless stated otherwise. Proteins were searched against an *L. longipalpis* protein sequence database containing 10,110 protein sequences from the VectorBase protein database and common contaminants, besides their respective reverse sequences, to estimate the false discovery rate (FDR). The carbamidomethylation of cysteine was set as a fixed modification, while methionine oxidation and N-terminal acetylation (protein) were allowed for as variable modifications. An FDR threshold of 0.01 was set for both the peptide and protein levels.

Protein quantification was performed using a label-free approach, where the peptide peaks were detected as three-dimensional features—retention time versus signal intensity (extracted ion chromatogram, XIC) versus mass–charge—and were aligned and compared across the runs, as previously described [13].

### 2.4. Metaproteomic Analysis

#### 2.4.1. Peptide Spectrum Matching (PSM)

The data analysis was performed using the PatternLab for Proteomics V (PLV-5.0.0.192) software, which is freely available at http://www.patternlabforproteomics.org/, (accessed on 17 January 2025) [14]. The protein sequences from *L. longipalpis* were retrieved from the UniProt database. The bacterial dataset from NCBI’s UniProtKB/Swiss-Prot database was downloaded on 17 January 2025, containing 336,569 protein sequences representing 3342 bacterial species. These datasets were integrated for analysis, and a target-decoy database was subsequently generated, incorporating reversed sequences and 123 common mass spectrometry contaminants. The data were preprocessed using the Y.A.D.A. 3.0 deconvolution algorithm to enable multiplexed spectrum identification. The mass spectra were identified using the Comet 2021 search engine, with search parameters including fully and semi-tryptic peptide candidates with masses ranging from 500 to 6000 Da, allowing for up to two missed cleavages. The precursor mass tolerance was set to 35 ppm, and MS/MS bins were set to 0.02 *m*/*z*. The modifications considered included the carbamidomethylation of cysteine as a fixed modification and the oxidation of methionine as a variable modification.

#### 2.4.2. Validation of PSMs

The validity of the PSMs was assessed using the Search Engine Processor (SEPro) [15]. Identifications were categorized by charge state (2+ and ≥3+) and by tryptic status, resulting in four distinct subgroups. For each subgroup, the XCorr, DeltaCN, DeltaPPM and Peak Match values were used to generate a Bayesian discriminator. The identifications were ranked in non-decreasing order based on the discriminator score, and a cutoff score was applied to maintain a false discovery rate (FDR) of 2% at the peptide level, as determined by the number of decoy hits [16]. This procedure was independently performed for each data subset to ensure an FDR independent of charge state or tryptic status. Additionally, filters were applied to retain only identifications with a minimum sequence length of five amino acid residues and a protein score greater than 2. Finally, PSMs deviating by more than 10 ppm from the theoretical mass were discarded. These stringent filtering steps ensured that the final protein-level FDR remained below 1% across all search results [17]. A given species was considered present in the sample when it presented at least one protein with at least 2 peptides related to a minimum of 5 MS1 scans.

#### 2.4.3. RNA Isolation from *N. umbratilis*

The intestines from 10 insects were separated from the carcasses. Three pools of 10 intestines and three pools of 10 carcasses were subjected to the RNA isolation process using the TRIzol^TM^ Reagent protocol (Invitrogen, Calrlsbad, CA, USA), following the manufacturer’s recommendations. The RNAs obtained were diluted in 20 μL of RNase-free water, quantified in NanoDrop and stored at −80 °C until use.

#### 2.4.4. cDNA Synthesis

Reverse transcriptase reactions were carried out using the GoScript^TM^ Reverse transcription system Kit (Promega, Madison, WI, USA) and The Applied Biosystems StepOne equipment (Applied Biosystems, Foster City, CA, USA), following the manufacturer’s recommendations. After synthesis, the samples were stored at −20 °C.

#### 2.4.5. Real-Time qPCR

qPCR was performed for quantitative analyses of gene expression using the SYBR^®^ Green PCR Master Mix (Applied Biosystems, Foster City, CA, USA) following the manufacturer’s recommendations. Reactions were performed in 96-well plates using the StepOnePlus Real-Time PCR Systems equipment (Applied Biosystems, Foster City, CA, USA). The real-time PCR program steps were as follows: 95 °C for 10 min, 40 cycles at 95 °C for 15 s and 60 °C for 1 min.

Primers for the quantification of SL9B2, PATH, PEPT1-LIKE, Paramyosin, SARA, CYSPC, Cytochrome P450, XPR1 and the reference gene GAPDH (Table 1) were designed based on the information and tools present on platforms such as NCBI Gene Bank, BLASTP and Vector Base. All qPCR reactions were performed in triplicate, and Ct values were measured.

### 2.5. Statistical Analysis

The differentially expressed proteins were obtained in Perseus, in which Student’s *t*-test corrected by a Benjamini–Hochberg FDR of 0.1 was applied. The results obtained in proteomics and PCR experiments were analyzed using the GraphPad Prisma 9.5.0. The statistical test used was the Ordinary one-way ANOVA [18]. Significance was given according to the following proportion: * *p* < 0.05; ** *p* < 0.01; *** *p* < 0.001; **** *p* < 0.0001.

## 3. Results

### 3.1. N. umbratilis Gut Proteome Analysis

**Figure 1:**
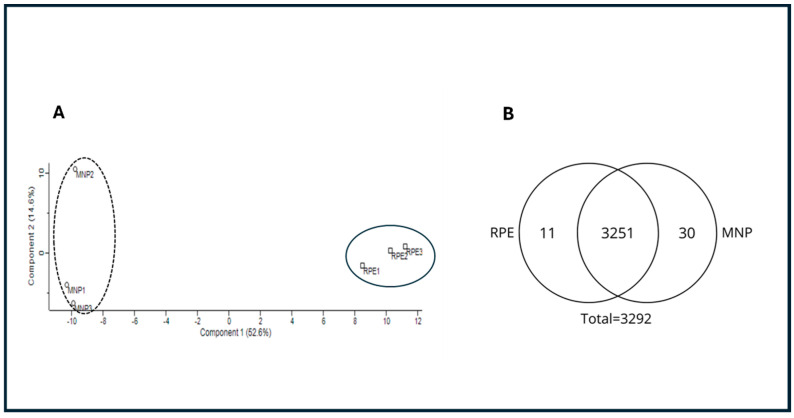
Proteomic comparison of *N. umbratilis* from MAN and RPE. (**A**) Principal component analysis (PCA) of gut proteomes from MAN and RPE populations. Distinct clustering indicates separation between two groups. (**B**) Venn diagram illustrates qualitative distribution of detected proteins. Total of 3292 proteins were identified, with 3251 shared between populations, 11 unique to RPE and 30 exclusive to MAN.

The *N. umbratilis* captured in multiple incursions in the municipalities of MAN and RPE were dissected, and the gut proteins were subjected to mass spectrometry analysis. A principal component analysis (PCA) indicated that, despite the higher similarity between populations, sample triplicates were consistent, confirming reproducibility. The analysis also revealed distinct characteristics separating the two populations (Figure 1A).

Qualitative analysis identified 3292 proteins, with 3251 detected in both populations. Additionally, 11 proteins were unique to RPE, while 30 were exclusive to MAN (Figure 1B).

### 3.2. Differentially and Exclusively Identified Proteins

**Figure 2:**
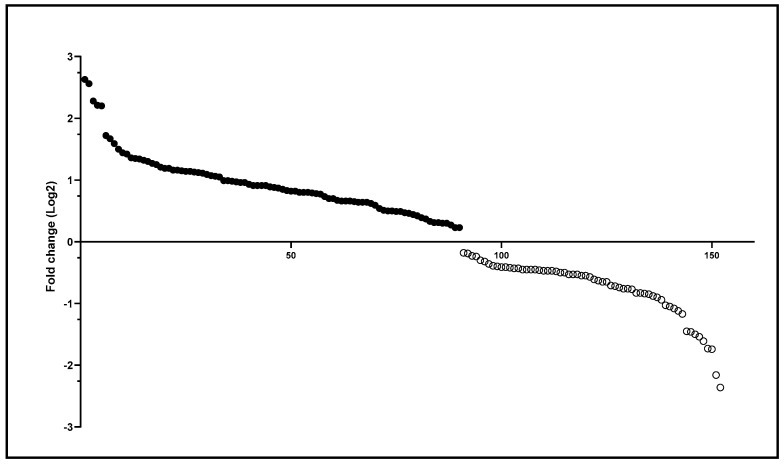
Differentially identified proteins in *N. umbratilis* from MAN and RPE. Scatter plot of differentially detected proteins based on Log2 fold-change values. Positive values (filled circles) indicate proteins more abundant in MAN, while negative values (empty circles) represent proteins more abundant in RPE.

Although qualitative variation was minimal, the MAN and RPE populations exhibited quantitative differences in intestinal protein content. A total of 152 differentially expressed proteins were identified (Table S1). The insects from MAN presented the highest number of differentially identified proteins, 90, with fold-change Log2 (FC) ranging from 0.23 to 2.63, while the *N. umbratilis* collected in RPE had 62 differentially detected proteins, with FC ranging from 0.18 to 2.36 (Figure 2).

Among the identified proteins, eight presented a potential role in the vectorial differences between the MAN and RPE populations. Four of them were more present in MAN. Three are related to the transport of protons and amino acids, listed here in decreasing order of FC: oligopeptide transport protein family 1 (PEPT1) (FC = 2.2); Proton-coupled amino acid transporter-like protein pathetic (PATH) (FC = 1.72) and Solute carrier family 9 member B2 (SL9B2 or Nha2) (FC = 1.34). The fourth protein was Paramyosin (FC = 1.44) related to intestinal motility. PATH is involved in the transport of amino acids coupled to proton influx and is primarily located on the surface of the intestinal epithelium and in lysosomal membranes [19]. PEPT1-like is a transporter located in the apical membrane of the intestinal absorptive epithelium, participating in the absorption of free amino acids and oligopeptides generated by the luminal digestion of proteins [20]. SL9B2 is a protein that catalyzes the antiport of sodium ions with extracellular protons and has an action in alkaline homeostasis due to its greater expression in intestinal epithelial tissue [21]. The activity of these proteins linked to the transport of amino acids and protons may also be related to an intestinal alkalinization process. Another differentially more abundant protein in MAN was Paramyosin, which can be found in the thick filaments of the muscle fibers of invertebrates and is related to intestinal motility [22]. The other group studied was composed of four proteins uniquely identified in MAN. These were as follows: Smad Anchor for Receptor Activation (SARA), Xenotropic and Polytropic Retrovirus Receptor 1 (XPR1), Calcium-dependent cytoplasmic cysteine proteinases, papain-like (CysPc) and Cytochrome P450.

SARA interacts with Suppressor of Mothers Against Decapentaplegic Homolog proteins (Smad), which mediate TGF-beta signaling from transmembrane serine–threonine receptor kinases to the cell nucleus. SARA recruits Smad to TGF-beta receptors for phosphorylation, thus playing a very important role in the immune system of sand flies [23,24]. XPR1 is a protein in which the domain is found at the amino terminus of a variety of proteins. The N terminus of the human XPR1 protein binds directly to the beta subunit of the G protein heterotrimer, leading to increased cAMP production. These findings suggest that all members of this family are involved in G-protein-associated signal transduction, thus being an important retroviral receptor in the activation of the second signal of cell metabolism and potentially affecting the parasite–vector relationship [25].

Another selected protein, CysPc, is a calcium-dependent cytoplasmic cysteine proteinase. It acts in the processes of cytoskeleton remodeling, cell differentiation, apoptosis and signal transduction, thus influencing several cellular events that may alter the conditions for *Leishmania* to establish itself in the digestive tract of sand flies [26]. Finally, Cytochrome P450 belongs to the insect and crustacean protein families, including the CYP6, CYP9 and CYP310 subfamilies, which are involved in insect hormone metabolism and xenobiotic detoxification [27], and this resistance may also change the intestinal conditions of insects, which would make it difficult for parasites to establish themselves.

### 3.3. Putative Protein–Protein Interaction Networks of Differentially Identified Peptides

**Figure 3:**
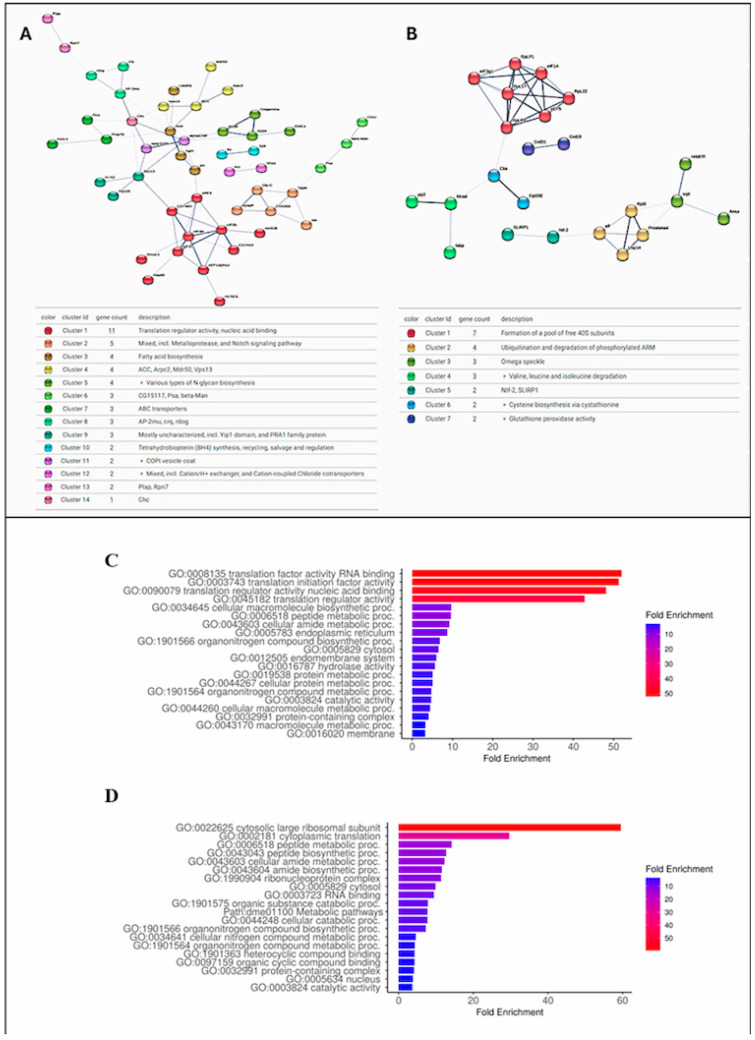
Analysis of interaction and gene ontology of proteins differentially identified (FC ≥ 0.5) in populations of MAN and RPE. (**A**,**B**)—Protein interaction maps of insects from MAN and RPE, respectively. Nodes with same color indicate proteins that form same cluster. (**C**,**D**)—Graphical charts of gene ontology fold enrichment analysis on ShinyGO 0.80 site of MAN and RPE proteins, respectively.

Among the 152 proteins differentially identified, those that presented FC (Log2) values equal to or greater than 0.5 were selected to be submitted to Protein–Protein Interaction Networks Functional Enrichment Analysis on the STRING website (https://cn.string-db.org/, accessed on 20 February 2025) [28]. Protein interactions from both populations were compared with the *Drosophila melanogaster* protein interactions database (NCBI taxonomy Id: 7227) (Figure 3A,B; Table S2A,B).

The intestinal proteome of the *N. umbratilis* population from MAN presented 74 proteins with an FC (Log2) greater than or equal to 0.5. Among these, 70 homologs were identified in the *D. melanogaster* interaction data available in the STRING database, with 49 showing interactions with each other with a protein–protein interaction (PPI) enrichment *p*-value of 1.7 × 10^−12^. The intestinal proteome of the RPE population presented 38 proteins with an FC (Log2) ≥ 0.5; of these, 23 showed interactions with each other with a PPI enrichment *p*-value of 8.3 × 10^−4^. This indicates that the proteins in each group have more interactions with each other than would be expected in a group of random proteins. The *N. umbratilis* homologs identified in the *D. melanogaster* genome on the STRING website were used in a gene ontology analysis on the ShinyGO 0.80 website (http://bioinformatics.sdstate.edu/go/, accessed on 20 February 2025) [29] (Figure 3C,D). The STRING and ShinyGo analyses showed that the majority of the enriched proteins in each group are related to each other and that despite being different between the groups, they are mainly related to RNA binding and gene translation.

### 3.4. Transcriptional Levels of Genes Coding for Proteins Differentially and Exclusively Identified in N. umbratilis from MAN

To investigate some of the differentially abundant proteins in MAN more thoroughly, we focused on four proteins with a fold-change (Log2) > 1.0 and the four uniquely detected in MAN. We performed semiquantitative PCR assays to measure the transcriptional levels of the genes coding for these proteins in the *N. umbratilis* of MAN and RPE.

Among the four proteins selected with an FC greater than one, three are related to protein and amino acid transport and gut motility: PEPT1, PATH, SLC9B2 and Paramyosin (Table S1). The selected four proteins uniquely detected in MAN were SARA, XPR1, CysPc and Cytochrome P450. These proteins are involved in the immune response modulation control of oxidative stress, the transcription and translation of proteins and xenobiotics detoxification [23,25,30,31].

**Figure 4:**
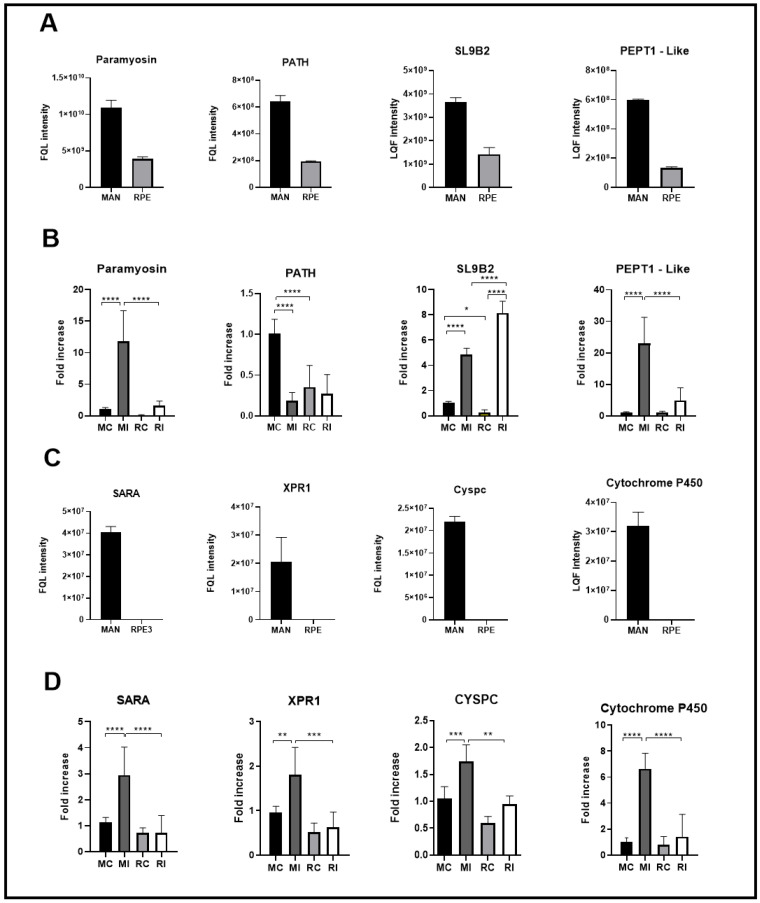
Quantitative and transcriptional analysis of gut proteins in *N. umbratilis* from MAN and RPE. (**A**) Relative abundance of proteins with fold-change (Log2) >1.0 in *N. umbratilis* from MAN compared to RPE. (**B**) Transcriptional levels of genes encoding proteins shown in (**A**), comparing *N. umbratilis* from MAN and RPE. (**C**) Relative abundance of proteins exclusively detected in *N. umbratilis* from MAN. (**D**) Transcriptional levels of genes encoding proteins exclusively identified in MAN. MC—MAN carcass; MI—MAN intestine; RC—RPE carcass; RI—RPE intestine. (* *p* < 0.05; ** *p* < 0.01; *** *p* < 0.001; **** *p* < 0.0001).

Figure 4 shows the transcriptional profile of the genes coding for proteins differentially and uniquely identified in MAN compared to the transcriptional profile of the same genes in RPE insects. All genes coding for proteins uniquely detected in the MAN gut proteome had their highest transcription levels in the gut of MAN insects (Figure 4C,D). Regarding the genes of the proteins differentially detected in MAN, we observed two exceptions: the PATH gene was more expressed in the carcass of insects from MAN and the SLB9 gene, which, despite having a significantly high expression in the intestine of *N. umbratilis* from MAN, presented higher transcriptional levels in the gut of RPE insects (Figure 4A,B).

The semiquantitative PCR results corroborated the mass spectrometry proteomic findings, reinforcing that *N. umbratilis* populations from MAN and RPE exhibit distinct molecular phenotypes in terms of intestinal protein composition.

### 3.5. Metaproteomic Analysis

**Figure 5:**
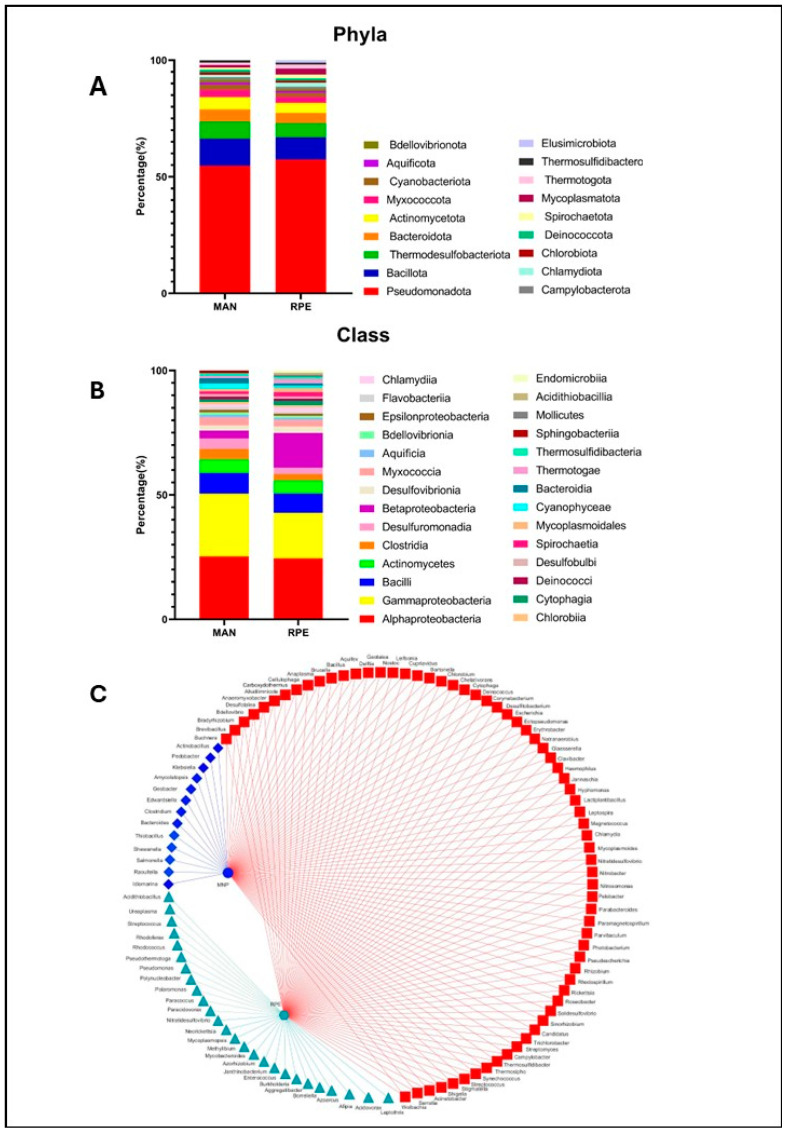
Gut microbiota composition of *L. umbratilis* from MAN and RPE. (**A**) Quantitative distribution of bacterial taxon phylum in intestinal microbiota of MAN and RPE *N. umbratilis* populations. (**B**) Quantitative distribution of bacterial taxon class in intestinal microbiota of MAN and RPE *N. umbratilis* populations. (**C**) Network diagram showing bacterial genera identified in intestinal microbiota. Red squares represent genera shared between both populations, blue diamonds represent genera unique to MAN (blue circle) population and green triangles represent genera unique to RPE (green hexagon) population.

Mass spectrometry proteomics and the real-time PCR assays showed that the *N. umbratilis* from the MAN and RPE populations present different molecular phenotypes in the expression of their intestinal proteins. These findings suggest differences in gut environmental conditions between the two populations. To try to shed light on this, by taking advantage of the proteomic data, which proved to be representative, we decided to mine the metaproteomic data regarding the composition of the bacterial flora in the gut of these insects. The microbiota of the MAN and RPE *N. umbratilis* populations was investigated by combining the NCBI UniProtKB/Swiss-Prot bacteria database, with 336,569 sequences covering 3342 species, with the *L. longipalpis* database. We detected a total of 1840 proteins belonging to 131 bacterial species present in the sand fly digestive tract, where 79 were shared between both populations, 16 were detected only in MAN and 36 were unique to RPE (Figure 5C; Table S3). The high number of species identified might be due to the fact that bacteria found in at least one of the replicates were taken into account.

The identified bacterial genera were distributed across 18 phyla and 28 classes. In terms of phyla, the microbiota of the MAN and RPE populations showed similar overall profiles, with a predominance of the phylum Pseudomonadota, synonym Proteobacteria, in both groups (54.73% in MAN and 57.39% in RPE). The other most abundant phyla were as follows: Bacillota, synonym Firmicutes (11.57% in MAN and 9.56% in RPE); Thermodesulfobacteriota (7.36% in MAN and 6.08% in RPE) and Bacteroidota, previously named Bacteroidetes, and Actinomycetota, both with 5.26% in MAN and 4.34% in RPE (Figure 5A).

A similar pattern was observed at the class level, with Alphaproteobacteria (25.26% in MAN and 24.5% in RPE); Gammaproteobacteria (25.26% in MAN and 18.26% in RPE); and Bacilli and Actinomycetes, both with 8.31% in MAN and 7.81% in RPE, being the most abundant. The populations of both locations presented similar percentages of each of the identified classes. The exception was Betaproteobacteria, which was more prevalent in RPE with 13.9% against 3.15% in MAN (Figure 5B).

Among the bacteria identified only in MAN (Figure 5C), an area with a low incidence of leishmaniasis, two species, *R. terrigena* and *Shewanella* sp., were previously described as having parasiticidal properties. The treatment of tomato plants infested with the parasitic nematode *Meloidogyne incognita* with *R. terrigena* eliminated the nematode infestation [9]. Cultures of *Trypanosoma cruzi* trypomastigotes treated with a crude extract of *Shewanella* sp. presented an inhibitory index (IC_50_) of 15 mg/mL [10].

## 4. Discussion

Data from the Brazilian Ministry of Health reveal that the municipalities of RPE and MAN in the state of Amazonas-BR have different leishmaniasis epidemiological conditions, with the first being considered endemic and the second non-endemic for the disease (https://www.gov.br/saude/pt-br/assuntos/saude-de-a-a-z/l/lt/situacao-epidemiologica, accessed on 12 September 2024).

Comparative genotypic and phenotypic analyses of *N. umbratilis* from MAN, RPE and Recife-PE, situated approximately 5000 Km from the Amazonian localities, revealed that, despite the distance, individuals from RPE are more closely related to insects from Recife than to sand flies from MAN [32]. Recent phylogenetic studies revealed that *N. umbratilis* from MAN and RPE belong to two distinct monophyletic evolutionary clades [6].

Ex vivo *L. guyanensis* adhesion experiments to the vectors’ guts showed that the parasites adhered significantly more to the intestine of insects originating from RPE than MAN [7]. In some vector–parasite pairs, the adhesion to the intestinal epithelium is an important step for the success of infection [33,34]. An important condition for the transmission of the etiological agent of ATL is the transformation of the pathogen aflagellate amastigote into infective metacyclic flagellate forms. This entire process takes place inside the vector’s digestive tract [35]. In this environment, the parasite interacts with several vector molecules and the microorganisms residing there. The success of the colonization of the vector’s digestive tract depends on the environmental conditions that the pathogen will encounter. These conditions are determined by the joint action of vector molecules and the resident microbiota [36,37].

Here, we carried out a comparative proteomic and metaproteomic analysis of the dissected guts of *N. umbratilis* from MAN and RPE. The results of the proteomic analysis revealed that despite great similarity, *N. umbratilis* from MAN and RPE can be divided into two distinct groups. This observation is in line with genotypic studies that analyzed polymorphisms in the Cytochrome C Oxidase (COI) gene in both populations [6,32].

In the comparative analysis, we observed that diverse proteins were differentially detected in each group. The gene ontology analysis showed that despite being distinct, these proteins are mainly related to protein synthesis activity in both groups. When we studied in more depth the eight proteins most detected or exclusively seen in sand flies from MAN, which is considered the population most refractory to *L. guyanensis* transmission, we noticed that of the four proteins selected among the most abundant in MAN, three (PEPT1, PATH and SL9B2) are related to amino acid and proton transport and are also involved in the control of intestinal pH. The fourth, Paramyosin, is involved in gut muscle contractibility. We also investigated the transcriptional levels of genes coding for the identified proteins SARA, XPR1, CysPc and Cytochrome P450, which were exclusively detected in MAN.

SARA plays a regulatory role in the TGF-β signaling pathway by interacting with Smad proteins, mediating signal transduction from transmembrane serine–threonine receptor kinases to the nucleus. It facilitates Smad recruitment to TGF-β receptors, leading to pathway activation through Smad phosphorylation. However, SARA can also inactivate TGF-β signaling by recruiting the catalytic subunit of protein phosphatase 1 (PP1c) [23,38,39]. The inhibition of the TGF-B pathway by gene silencing via RNAi or by use of the inhibitor SB431542 resulted in a reduction in *L. i. chagasi* survival in sand flies, suggesting that under natural conditions, the parasite benefits from the insect LlTGF-β pathway [24]. The high levels of the SARA protein in *N. umbralis* from MAN may be related to the greater inhibition of the TGF-β pathway and consequently a greater refractoriness to *L. guyanensis* infection.

The N-terminus of the human XPR1 protein binds directly to the beta subunit of the G protein heterotrimer, leading to increased cAMP production. Studies have shown that increased cAMP production leads to an increased alkalinization of the intestine of *L. longipalpis* [40,41]. These findings suggest that all members of this family are involved in G-protein-associated signal transduction, making it an important retroviral receptor in activating secondary signaling pathways in cell metabolism and potentially influencing the parasite–vector relationship [25].

Another selected protein, CysPc, is a calcium-dependent cytoplasmic cysteine proteinase. It participates in cytoskeleton remodeling, cellular differentiation, apoptosis and signal transduction, thus influencing several cellular events that can alter the conditions for *Leishmania* to establish itself in the digestive tract of sand flies [26].

Finally, Cytochrome P450 belongs to the families of insect and crustacean proteins, including the CYP6, CYP9 and CYP310 subfamilies. These are involved in insect hormone metabolism and xenobiotic detoxification [27], which may further change the intestinal conditions of the insects, creating difficulties for the parasites to establish themselves.

All these relationships between proteins and their impact on parasite transmission remain hypothetical and require further studies for a better understanding.

Through the qPCR experiments, we saw that the mRNA molecules that encode SL9B2 are more abundant in the intestine of both populations compared to their carcasses (Figure 5A), which was expected due to the action of this protein in the epithelial tissue of the insect’s digestive tract [21].

We also observed that the carcass from MAN has a higher relative expression of the SL9B2 gene than that from RPE, which is at odds with the proteomic results. This might be due to some post-transcriptional regulatory factor, which leads to the need for further studies to better understand the influence and action of this protein on sand flies.

PATH is involved in the absorption of amino acids such as alanine, functioning in symport with the influx of protons and the absorption of proline. It also plays a role in the positive modulation of cell growth by regulating the TOR pathway (especially in neurons). This protein family is primarily located in the surface epithelium of the intestine or in the lysosome membrane, highlighting its role in the intestinal absorption of amino acids and their reabsorption after the intralysosomal digestion of proteins [19]. As it is a protein that translocates amino acids in symport with protons, its activity must contribute to the alkalinization of the intestine. This mechanism has been previously described in a protein with similar activity in the intestine of *L. longipalpis* [40].

SLC9B2, cation proton antiporter 2, is a protein that catalyzes the antiport of sodium ions with extracellular protons. The greater abundance of this protein in the large intestinal epithelium and Malpighian tubules suggests a role in ionic homeostasis. The increased expression of SLC9B2 in some organs of the digestive tract and the carcass of *D. melanogaster* was observed after the induction of salt stress [21]. The predominance of this protein in the MAN population may be another indication that their intestinal lumen has a more basic pH, since the activity of the protein sequesters protons from the environment. Additionally, the secretion of sodium ions into the intestinal lumen mediated by SLC9B2 may contribute to a more hypertonic environment, potentially affecting *Leishmania* during infection or influencing the local microbiota.

In mammals, tPEPT1 is a transporter located in the apical membrane of the intestinal absorptive epithelium, participating in the absorption of free amino acids and short-chain peptides generated by the luminal digestion of proteins [20]. PTR family transporters, such as PEPT1, catalyze the translocation of their substrate together with protons in the symport model [42]. Its greater expression in the MAN population is an indication that there may be a greater influx of protons in the epithelial cells of the sand fly’s intestine mediated by the action of this transporter.

It has previously been observed that Leishmania demonstrates greater efficiency in the in vitro metacyclogenesis process when cultivated in a culture medium with an acidic pH [43,44]. Therefore, the predominance of proteins associated with intestinal lumen alkalinization in the insect population lacking vector competence for *L. guyanensis* could present a challenge for the parasites to efficiently develop in the vertebrate infective stages, potentially contributing to the reduced vectorial capacity of this population.

Another discussion point that can be raised from these data is the possible relevance of the complement system proteins ingested with the blood bolus of the vertebrate host in the success of the establishment and development of the parasite in the vector’s intestine. It was demonstrated that the sensitivity of the promastigote forms of *Leishmania* from the subgenus *Viannia*, such as *L.* (*V.*) *panamensis*, *L.* (*V.*) *braziliensis* and also *L.* (*V*.) *guyanensis*, to the lysis mediated by the alternative pathway of the system complement remains high during all stages of development when compared to the *Leishmania* subgenus [45]. However, the activity of the alternative pathway of the complement system is pH-dependent [46]. Therefore, the complement system proteins ingested with the blood bolus may differently influence the fitness of the parasites when exposed to the luminal pH of these two populations. However, as the optimal activity of the alternative complement system occurs within a pH window, it would be necessary to experimentally investigate this aspect to determine which population offers an intestinal environment that is less conducive to the action of the alternative pathway of the complement system.

Another finding that drew our attention is the fact that, when comparing the two populations, it was observed that MAN sand flies expressed more muscle filament component protein. As it is an intestinal proteome, the first question that arises is whether this difference in the structural composition of the heavy chains of muscle filaments can reflect a difference in intestinal motility. The literature describes that some *Leishmania* species secrete virulence factors capable of inhibiting the peristalsis of the mid and/or posterior intestine of their invertebrate vectors, making it difficult to expel the parasites in the fecal bolus and contributing to the persistence of the infection [47,48]. Perhaps the greater presence of Paramyosin in the intestinal epithelium of *N. umbratilis* is related to greater resistance to the action of inhibiting the intestinal peristalsis of these insects, which would make it difficult for the parasites to adhere to the intestinal wall of the vectors.

In sand flies, the entire development of the Leishmania parasite occurs within the digestive tract, causing the pathogen to coexist closely with the other occupants of the intestinal milieu. The set of microorganisms that make up the sand fly’s gut microbiota, which includes viruses and fungi, is predominantly made up of bacteria. Several studies have revealed the important role of sand fly intestinal bacteria in the gut colonization, development and transmission of parasites [49,50,51,52,53]. Sand fly larvae fed with food based on sterilized rabbit feces grew less and had lower hatched rates, and these adults laid fewer eggs than insects whose larvae were fed with non-sterile food [54]. Other studies have also highlighted the important role of the intestinal microbiota in the development and transmission of Leishmania parasites [55,56,57]. We observed that insects from both localities presented a similar bacteriome composition. Interestingly, the phyla and genera most identified in *N. umbratilis* from MAN and RPE in northern Brazil were the same as those found in sand flies from three endemic ZVL foci in the northeastern (Bojnord in North Khorasan Province), northwestern (Meshkinshar in Ardabil Province) and southwest (Mamasani in Fras Province) regions of Iran, and the phyla Proteobacteria and Firmicutes and the genus Pseudomonas were among the most abundant in the locations studied [58]. The presence of the genus Pseudomonas was observed in different sand fly species from field collections or laboratory-reared colonies, in both the New and Old Worlds [59]. The phyla Proteobacteria and Firmicutes have many species with diazotrophic capacity, which can fix atmospheric oxygen in other molecules. The presence of these bacteria would be advantageous for the vector [60]. Perhaps this advantageous relationship could explain the prevalent presence of these phyla among sand fly species. Despite the limited scope of our metaproteomic data obtained from the mass spectrometric analysis of intestinal proteins from *N. umbratilis*, we can observe differences in the bacterial composition of the microbiota of sand flies from both locations. Recent work has demonstrated the deleterious role of two different bacterial commensals, *Delftia tsuruhatensis* and *Serratia ureilytica*, on the transmission of plasmodia by anopheline mosquitoes [52,53]. Here, we identified two bacteria exclusive to the MAN microbiota that were described as having parasiticidal proprieties, the bacteria *R. terrigena*, able to kill nematodes infesting tomato plants, and *Shewanella* sp, which inhibited the development of protozoan parasite *T. cruzi* in culture [9,10]. This demonstrates the importance of new studies regarding the composition of the microbiota of MAN and RPE sand fly vectors. Perhaps the scenario formed by the activity of the most abundant proteins in MAN, associated with the presence of microorganisms harmful to parasites, contributes to the epidemiological differences presented by these two locations. Although backed up by data from the literature, the hypotheses put forward here to explain the vector differences observed between the two populations are speculations. Further studies are needed to confirm them.

## Figures and Tables

**Table 1:** Primers.

Primer	Sequence 5′–3′
GAPDH-F	TTC GCA GAA GAC AGT GATG G
GAPDH-R	CCC TTC ATC GGT CTG GAC TA
SL9B2-F	AGCCACCGACCATCCCT
SL9B2-R	TTTGTGACCGTGAAGACG
PATH-F	AACTACACCATGAGGAC
PATH-R	TCCCAGTACGTGACCAT
PEPT1-LIKE-F	CCTCCAAGATATACACGATGATT
PEPT1-LIKE-R	CTCCCAATCTCAATTCATA
PARAMYOSIN-F	ATCGACAGGAGGCGGAAG
PARAMYOSIN-R	TTCAATCCTCTGACGAAG
SARA-F	ACTACCGCTACTACCCGAG
SARA-R	CACGAATGGATGAAAG
XPR1-F	CTACTACTTTGCCATT
XPR1-R	CCAGAGGAGCCGTTAT
CYSPC-F	TGCTGAGAATCCACAGTTT
CYSPC-R	CCTTCCTGCCATCATT
CYTOCHROME P450-F	TAGGAAGATGGGAAGGAAG
CYTOCHROME P450-R	CTTTATCCACTGTTTTCAC

## Data Availability

The original contributions presented in this study are included in this article/Supplementary Materials.

## Supplementary Materials

The following supporting information can be downloaded at: https://www.mdpi.com/article/10.3390/microorganisms13061304/s1, Table S1: Differentially identified proteins in MAN (positive values) and RPE (negative values). Table S2: (A) STRING MCL MAN clusters and their proteins. (B) STRING MCL RPE clusters and their proteins. Table S3: Bacteria identified in MAN and RPE.
