# Supplementary material for: Comparative Gut Proteome of Nyssomyia umbratilis from Leishmaniasis Endemic and Non-Endemic Areas of Amazon Reveals Differences in Microbiota and Proteins Related to Immunity and Gut Function
Source: Microorganisms. 2025 Jun 4;13(6):1304. doi: 10.3390/microorganisms13061304 (PMC12195068; doi:10.3390/microorganisms13061304)
Supplement: Supplementary file 1 [file microorganisms-13-01304-s001.zip › Table S3.pdf]

Bact. MAN Exclusive		Bact. RPE Exclusive		Bact. MAN & RPE	
Acinetobacter sp. Tol 5	https://www.ncbi.nlm.nih.gov/protein/127525	Rhodococcus rhodochrous	https://www.ncbi.nlm.nih.gov/protein/128169	Cellulophaga lytica	https://www.ncbi.nlm.nih.gov/protein/114545
Actinobacillus pleuropneumoniae serovar 3 str. JL03	https://www.ncbi.nlm.nih.gov/protein/543995	Pseudomonas fluorescens	https://www.ncbi.nlm.nih.gov/protein/585710	Rhodospirillum rubrum	https://www.ncbi.nlm.nih.gov/protein/114564
Amycolatopsis methanolica	https://www.ncbi.nlm.nih.gov/protein/729048	Aggregatibacter actinomycetemcomitans	https://www.ncbi.nlm.nih.gov/protein/2492494	Pseudescherichia vulneris	https://www.ncbi.nlm.nih.gov/protein/120642
Bacillus subtilis subsp. subtilis str. 168	https://www.ncbi.nlm.nih.gov/protein/1168238	Escherichia coli K-12	https://www.ncbi.nlm.nih.gov/protein/2506132	Serratia marcescens	https://www.ncbi.nlm.nih.gov/protein/129141
Bacteroides fragilis YCH46	https://www.ncbi.nlm.nih.gov/protein/3914215	Ureaplasma parvum serovar 3 str. ATCC 700970	https://www.ncbi.nlm.nih.gov/protein/13959349	Photobacterium damselae subsp. piscicida	https://www.ncbi.nlm.nih.gov/protein/728805
Bartonella bacilliformis KC583	https://www.ncbi.nlm.nih.gov/protein/3914225	Sinorhizobium meliloti 1021	https://www.ncbi.nlm.nih.gov/protein/24211682	Stigmatella aurantiaca	https://www.ncbi.nlm.nih.gov/protein/1168582
Bradyrhizobium diazoefficiens USDA 110	https://www.ncbi.nlm.nih.gov/protein/3915692	Bradyrhizobium diazoefficiens USDA 110	https://www.ncbi.nlm.nih.gov/protein/30179449	Haemophilus influenzae Rd KW20	https://www.ncbi.nlm.nih.gov/protein/1169791
Clostridium kluyveri DSM 555	https://www.ncbi.nlm.nih.gov/protein/31563263	Serratia sp. E-15	https://www.ncbi.nlm.nih.gov/protein/32172419	Mycoplasmoides pneumoniae M129	https://www.ncbi.nlm.nih.gov/protein/2493019
Edwardsiella ictaluri 93-146	https://www.ncbi.nlm.nih.gov/protein/47606093	Bradyrhizobium diazoefficiens USDA 110	https://www.ncbi.nlm.nih.gov/protein/68566296	Aquifex aeolicus VF5	https://www.ncbi.nlm.nih.gov/protein/3913127
Escherichia coli CFT073	https://www.ncbi.nlm.nih.gov/protein/55977752	Enterococcus faecalis V583	https://www.ncbi.nlm.nih.gov/protein/81437517	Chlamydia pneumoniae	https://www.ncbi.nlm.nih.gov/protein/6831543
Geobacter sulfurreducens PCA	https://www.ncbi.nlm.nih.gov/protein/81637588	Pseudomonas aeruginosa PAO1	https://www.ncbi.nlm.nih.gov/protein/81540583	Rickettsia prowazekii str. Madrid E	https://www.ncbi.nlm.nih.gov/protein/7387941
Idiomarina loihiensis L2TR	https://www.ncbi.nlm.nih.gov/protein/81821624	Neorickettsia sennetsu str. Miyayama	https://www.ncbi.nlm.nih.gov/protein/118573703	Buchnera aphidicola str. APS (Acyrthosiphon pisum)	https://www.ncbi.nlm.nih.gov/protein/11131985
Klebsiella pneumoniae 342	https://www.ncbi.nlm.nih.gov/protein/88942919	Alkalilimnicola ehrlichii MLHE-1	https://www.ncbi.nlm.nih.gov/protein/122311303	Sinorhizobium meliloti 1021	https://www.ncbi.nlm.nih.gov/protein/17380435
Pedobacter heparinus DSM 2366	https://www.ncbi.nlm.nih.gov/protein/118573774	Rhodoferax ferrireducens T118	https://www.ncbi.nlm.nih.gov/protein/122479525	Nostoc sp. PCC 7120 = FACHB-418	https://www.ncbi.nlm.nih.gov/protein/20141410
Raoultella terrigena	https://www.ncbi.nlm.nih.gov/protein/122064522	Borreliella afzelii PKo	https://www.ncbi.nlm.nih.gov/protein/123046963	Buchnera aphidicola str. Sg (Schizaphis graminum)	https://www.ncbi.nlm.nih.gov/protein/25008569
Salmonella enterica subsp. enterica serovar Typhi	https://www.ncbi.nlm.nih.gov/protein/166198436	Polaromonas sp. JS666	https://www.ncbi.nlm.nih.gov/protein/123355075	Nitratidesulfovibrio vulgaris str. Hildenborough	https://www.ncbi.nlm.nih.gov/protein/60390490
Serratia marcescens	https://www.ncbi.nlm.nih.gov/protein/226706712	Mycoplasmopsis synoviae 53	https://www.ncbi.nlm.nih.gov/protein/123775416	Acinetobacter sp.	https://www.ncbi.nlm.nih.gov/protein/75532470
Serratia marcescens	https://www.ncbi.nlm.nih.gov/protein/226731295	Acidovorax sp. JS42	https://www.ncbi.nlm.nih.gov/protein/150384505	Acinetobacter baylyi ADP1	https://www.ncbi.nlm.nih.gov/protein/81393896
Serratia marcescens	https://www.ncbi.nlm.nih.gov/protein/259511503	Paracidovorax citrulli AAC00-1	https://www.ncbi.nlm.nih.gov/protein/166198427	Anaplasma marginale str. St. Maries	https://www.ncbi.nlm.nih.gov/protein/81599150
Shewanella sp. MR-4	https://www.ncbi.nlm.nih.gov/protein/550540775	Candidatus Ruthia magnifica str. Cm (Calyptogena magnifica)	https://www.ncbi.nlm.nih.gov/protein/166199103	Leifsonia xyli subsp. xyli str. CTCB07	https://www.ncbi.nlm.nih.gov/protein/81612770
Thiobacillus denitrificans ATCC 25259	https://www.ncbi.nlm.nih.gov/protein/1915400776	Parvibaculum lavamentivorans DS-1	https://www.ncbi.nlm.nih.gov/protein/171769690	Lactiplantibacillus plantarum WCFS1	https://www.ncbi.nlm.nih.gov/protein/81631036
		Azorhizobium caulinodans ORS 571	https://www.ncbi.nlm.nih.gov/protein/172048044	Desulfotalea psychrophila LSv54	https://www.ncbi.nlm.nih.gov/protein/81643219
		Azoarcus olearius	https://www.ncbi.nlm.nih.gov/protein/187470734	Wolbachia endosymbiont of Drosophila melanogaster	https://www.ncbi.nlm.nih.gov/protein/81700105
		Burkholderia pseudomallei 1106a	https://www.ncbi.nlm.nih.gov/protein/187470738	Shigella flexneri	https://www.ncbi.nlm.nih.gov/protein/81724232
		Burkholderia vietnamiensis G4	https://www.ncbi.nlm.nih.gov/protein/189036643	Photobacterium profundum SS9	https://www.ncbi.nlm.nih.gov/protein/81828635
		Janthinobacterium sp. Marseille	https://www.ncbi.nlm.nih.gov/protein/189036669	Bdellovibrio bacteriovorus HD100	https://www.ncbi.nlm.nih.gov/protein/81829096
		Methylibium petroleiphilum PM1	https://www.ncbi.nlm.nih.gov/protein/189036677	Nitrosomonas europaea ATCC 19718	https://www.ncbi.nlm.nih.gov/protein/81838782
		Polynucleobacter asymbioticus QLW-P1DMWA-1	https://www.ncbi.nlm.nih.gov/protein/189036717	Rhodospirillum rubrum ATCC 11170	https://www.ncbi.nlm.nih.gov/protein/90110006
		Acidovorax sp. JS42	https://www.ncbi.nlm.nih.gov/protein/189044646	Anaeromyxobacter dehalogenans 2CP-C	https://www.ncbi.nlm.nih.gov/protein/115312234
		Polaromonas naphthalenivorans CJ2	https://www.ncbi.nlm.nih.gov/protein/189081766	Candidatus Pelagibacter ubique HTCC1062	https://www.ncbi.nlm.nih.gov/protein/115312260
		Streptococcus suis 98HAH33	https://www.ncbi.nlm.nih.gov/protein/190358731	Rhizobium johnstonii 3841	https://www.ncbi.nlm.nih.gov/protein/118573486
		Candidatus Endomicrobiellum trichonymphae	https://www.ncbi.nlm.nih.gov/protein/205829219	Brucella abortus 2308	https://www.ncbi.nlm.nih.gov/protein/118573512
		Acidithiobacillus ferrooxidans ATCC 53993	https://www.ncbi.nlm.nih.gov/protein/218534379	Carboxydothermus hydrogenoformans Z-2901	https://www.ncbi.nlm.nih.gov/protein/118573518
		Delftia acidovorans SPH-1	https://www.ncbi.nlm.nih.gov/protein/226704113	Jannaschia sp. CCS1	https://www.ncbi.nlm.nih.gov/protein/118573688
		Leptothrix cholodnii SP-6	https://www.ncbi.nlm.nih.gov/protein/226704147	Paramagnetospirillum magneticum AMB-1	https://www.ncbi.nlm.nih.gov/protein/118573694
		Nitratidesulfovibrio vulgaris str. 'Miyazaki F'	https://www.ncbi.nlm.nih.gov/protein/226741081	Cupriavidus pinatubonensis JMP134	https://www.ncbi.nlm.nih.gov/protein/118573728
		Chlorobium phaeobacteroides BS1	https://www.ncbi.nlm.nih.gov/protein/229558598	Nitrobacter hamburgensis X14	https://www.ncbi.nlm.nih.gov/protein/119366179
		Afipia carboxidovorans OM5	https://www.ncbi.nlm.nih.gov/protein/229890078	Leptospira borgpetersenii serovar Hardjo-bovis str. JB197	https://www.ncbi.nlm.nih.gov/protein/122280912
		Mycobacteroides abscessus ATCC 19977	https://www.ncbi.nlm.nih.gov/protein/238688915	Alkalilimnicola ehrlichii MLHE-1	https://www.ncbi.nlm.nih.gov/protein/122310604
		Candidatus Amoebophilus asiaticus 5a2	https://www.ncbi.nlm.nih.gov/protein/238692324	Shigella flexneri 5 str. 8401	https://www.ncbi.nlm.nih.gov/protein/123048396
		Pseudothermotoga lettingae TMO	https://www.ncbi.nlm.nih.gov/protein/254766143	Cytophaga hutchinsonii ATCC 33406	https://www.ncbi.nlm.nih.gov/protein/123059035
		[Acidovorax] ebreus TPSY	https://www.ncbi.nlm.nih.gov/protein/254783248	Hyphomonas neptunium ATCC 15444	https://www.ncbi.nlm.nih.gov/protein/123128756
		Clavibacter michiganensis subsp. michiganensis NCPPB 382	https://www.ncbi.nlm.nih.gov/protein/254798896	Erythrobacter litoralis HTCC2594	https://www.ncbi.nlm.nih.gov/protein/123293698
		[Acidovorax] ebreus TPSY	https://www.ncbi.nlm.nih.gov/protein/254808148	Synechococcus sp. CC9311	https://www.ncbi.nlm.nih.gov/protein/123327552
		Chlamydia trachomatis L2b/UCH-1/proctitis	https://www.ncbi.nlm.nih.gov/protein/259511500	Chelativorans sp. BNC1	https://www.ncbi.nlm.nih.gov/protein/123353123
		Acidovorax sp. JS42	https://www.ncbi.nlm.nih.gov/protein/263430659	Roseobacter denitrificans OCh 114	https://www.ncbi.nlm.nih.gov/protein/123362354
		Paracoccus denitrificans PD1222	https://www.ncbi.nlm.nih.gov/protein/1559988658	Rhizobium etli CFN 42	https://www.ncbi.nlm.nih.gov/protein/123513416
				Campylobacter fetus subsp. fetus 82-40	https://www.ncbi.nlm.nih.gov/protein/158512510
				Anaeromyxobacter sp. Fw109-5	https://www.ncbi.nlm.nih.gov/protein/166201650
				Parabacteroides distasonis ATCC 8503	https://www.ncbi.nlm.nih.gov/protein/166201723
				Rickettsia canadensis str. McKiel	https://www.ncbi.nlm.nih.gov/protein/166222408
				Thermosipho melanesiensis BI429	https://www.ncbi.nlm.nih.gov/protein/166222423
				Pelobacter propionicus DSM 2379	https://www.ncbi.nlm.nih.gov/protein/166223059
				Bacillus pumilus SAFR-032	https://www.ncbi.nlm.nih.gov/protein/166990354
				Ectopseudomonas mendocina ymp	https://www.ncbi.nlm.nih.gov/protein/166990361
				Bacillus cytotoxicus NVH 391-98	https://www.ncbi.nlm.nih.gov/protein/189081327
				Geotalea uraniireducens Rf4	https://www.ncbi.nlm.nih.gov/protein/189081335
				Streptococcus suis 05ZYH33	https://www.ncbi.nlm.nih.gov/protein/190358201
				Bartonella bacilliformis KC583	https://www.ncbi.nlm.nih.gov/protein/190358257
				Campylobacter fetus subsp. fetus 82-40	https://www.ncbi.nlm.nih.gov/protein/190358269
				Clavibacter michiganensis subsp. michiganensis NCPPB 382	https://www.ncbi.nlm.nih.gov/protein/190358280
				Brucella anthropi ATCC 49188	https://www.ncbi.nlm.nih.gov/protein/190358689
				Parvibaculum lavamentivorans DS-1	https://www.ncbi.nlm.nih.gov/protein/190358697
				Delftia acidovorans SPH-1	https://www.ncbi.nlm.nih.gov/protein/226699393
				Streptococcus pyogenes NZ131	https://www.ncbi.nlm.nih.gov/protein/226738185
				Delftia acidovorans SPH-1	https://www.ncbi.nlm.nih.gov/protein/226739868
				Chlorobium phaeobacteroides BS1	https://www.ncbi.nlm.nih.gov/protein/226740178
				Trichlorobacter lovleyi SZ	https://www.ncbi.nlm.nih.gov/protein/226740205
				Rhizobium etli CIAT 652	https://www.ncbi.nlm.nih.gov/protein/226740664
				Streptomyces griseus subsp. griseus NBRC 13350	https://www.ncbi.nlm.nih.gov/protein/226740678
				Acinetobacter baumannii AB307-0294	https://www.ncbi.nlm.nih.gov/protein/226741072
				Natranaerobius thermophilus JW/NM-WN-LF	https://www.ncbi.nlm.nih.gov/protein/229463771
				Brevibacillus brevis NBRC 100599	https://www.ncbi.nlm.nih.gov/protein/254767218
				Desulfitobacterium hafniense DCB-2	https://www.ncbi.nlm.nih.gov/protein/254778002
				Glaesserella parasuis SH0165	https://www.ncbi.nlm.nih.gov/protein/254807699
				Magnetococcus marinus MC-1	https://www.ncbi.nlm.nih.gov/protein/254808157
				Corynebacterium kroppenstedtii DSM 44385	https://www.ncbi.nlm.nih.gov/protein/259645832
				Solidesulfovibrio magneticus RS-1	https://www.ncbi.nlm.nih.gov/protein/259647190
				Deinococcus deserti VCD115	https://www.ncbi.nlm.nih.gov/protein/259647676
				Thermosulfidibacter takaii ABI70S6	https://www.ncbi.nlm.nih.gov/protein/1384039502
