# Supplementary material for: Comparative Gut Proteome of Nyssomyia umbratilis from Leishmaniasis Endemic and Non-Endemic Areas of Amazon Reveals Differences in Microbiota and Proteins Related to Immunity and Gut Function
Source: Microorganisms. 2025 Jun 4;13(6):1304. doi: 10.3390/microorganisms13061304 (PMC12195068; doi:10.3390/microorganisms13061304)
Supplement: Supplementary file 1 [file microorganisms-13-01304-s001.zip › Table S1.pdf]

**Protein IDs**	**Protein description**	**Fold-change MNP/RPE (log2) (positive values indicate increase in MNP)**
gi|813426044	NADH dehydrogenase subunit 1 (mitochondrion) [Nyssomyia umbratilis]	2,63
gi|1131310692	putative splicing factor 3b subunit 3, partial [Nyssomyia neivai]	2,56
gi|1131324606	putative beta-glucuronidase gusb glycosylhydrolase superfamily 2 [Nyssomyia neivai]	2,28
gi|1131304941	putative prenyl protease type i [Nyssomyia neivai]	2,21
gi|1131324926	putative h+/oligopeptide symporter [Nyssomyia neivai]	2,20
gi|1131309116	putative amino acid transporter [Nyssomyia neivai]	1,72
gi|1131317691	putative translation elongation factor ef-1 alpha/tu [Nyssomyia neivai]	1,67
gi|1131324290	putative dolichyl-diphosphooligosaccharide--protein glycosyltransferase subunit stt3b [Nyssomyia neivai]	1,59
gi|1131313631	putative camp-dependent protein kinase catalytic subunit pka, partial [Nyssomyia neivai]	1,50
gi|1131327191	Putative Myosin class ii heavy chain [Nyssomyia neivai]	1,44
gi|1131305175	putative conserved plasma membrane protein, partial [Nyssomyia neivai]	1,42
gi|1131321837	putative xanthine dehydrogenase [Nyssomyia neivai]	1,36
gi|1131316893	putative beta-mannosidase [Nyssomyia neivai]	1,35
gi|1131323303	putative mitochondrial sodium/hydrogen exchanger 9b2 isoform x1, partial [Nyssomyia neivai]	1,34
gi|1131324282	putative dolichyl-diphosphooligosaccharide--protein glycosyltransferase subunit stt3a [Nyssomyia neivai]	1,32
gi|1131323812	putative mitochondrial phosphate carrier protein [Nyssomyia neivai]	1,30
gi|1131310602	putative atp-dependent rna helicase, partial [Nyssomyia neivai]	1,27
gi|1131320369	putative vesicle coat complex copi alpha subunit [Nyssomyia neivai]	1,25
gi|1131317403	putative translation initiation factor 4f helicase subunit eif-4a, partial [Nyssomyia neivai]	1,21
gi|1131320201	putative vacuolar protein [Nyssomyia neivai]	1,19
gi|1131322879	putative ecdysteroid kinase [Nyssomyia neivai]	1,19
gi|1131319617	putative rab gtpase [Nyssomyia neivai]	1,16
gi|1131305229	putative conserved plasma membrane protein [Nyssomyia neivai]	1,16
gi|427188252	hypothetical protein [Lutzomyia ayacuchensis]	1,15

gi|1131305511	putative 50 kda midgut protein [Nyssomyia neivai]	1,14
gi|1131312821	putative plasma membrane glycoprotein cd36 [Nyssomyia neivai]	1,14
gi|1131324948	putative dipeptidyl aminopeptidase, partial [Nyssomyia neivai]	1,13
gi|1131323059	putative s-adenosylcysteine hydrolase [Nyssomyia neivai]	1,12
gi|1131313703	putative camp-dependent protein kinase catalytic subunit pka, partial [Nyssomyia neivai]	1,11
gi|1131318929	putative oligosaccharyltransferase gamma subunit [Nyssomyia neivai]	1,09
gi|1131326399	putative multidrug resistance-associated protein/mitoxantrone resistance protein [Nyssomyia neivai]	1,07
gi|1131326633	putative sorbitol dehydrogenase [Nyssomyia neivai]	1,06
gi|1131314331	putative mitogen-activated protein kinase mapk kinase mkk7/jnkk2, partial [Nyssomyia neivai]	1,05
gi|1131326609	putative dehydrogenase [Nyssomyia neivai]	0,99
gi|1131319537	putative peptide exporter abc superfamily protein [Nyssomyia neivai]	0,99
gi|1131318845	putative metalloproteinase-related collagenase pm5, partial [Nyssomyia neivai]	0,98
gi|1131318356	putative 26s proteasome regulatory complex subunit rpn7/psmd6 [Nyssomyia neivai]	0,97
gi|1131309138	putative amino acid transporter [Nyssomyia neivai]	0,96
gi|1131313261	putative hydroxysteroid 17-beta dehydrogenase 11, partial [Nyssomyia neivai]	0,96
gi|1131305217	putative unknown conserved protein [Nyssomyia neivai]	0,93
gi|1131320465	putative vesicle coat complex copi beta subunit [Nyssomyia neivai]	0,91
gi|1131324422	putative glycosyltransferase, partial [Nyssomyia neivai]	0,91
gi|1131316287	putative phospholipase a2-activating protein, partial [Nyssomyia neivai]	0,91
gi|1131317723	putative translation initiation factor 4f helicase subunit eif-4a [Nyssomyia neivai]	0,91
gi|1131326385	putative multidrug resistance-associated protein/mitoxantrone resistance protein [Nyssomyia neivai]	0,89
gi|1131310734	putative atp-dependent rna helicase [Nyssomyia neivai]	0,88
gi|1131316769	putative metallopeptidase family m24 [Nyssomyia neivai]	0,87
gi|1131322667	putative serine/threonine kinase receptor-associated protein [Nyssomyia neivai]	0,85
gi|1131318823	putative prolyl endopeptidase isoform x1 [Nyssomyia neivai]	0,83

gi|1131318825	putative metalloprotease [Nyssomyia neivai]	0,82
gi|1131308544	putative sulfate/bicarbonate/oxalate exchanger sat-1 [Nyssomyia neivai]	0,82
gi|1131323173	putative nadp transhydrogenase mitochondrial [Nyssomyia neivai]	0,80
gi|1131318841	putative tripeptidyl-peptidase 2 [Nyssomyia neivai]	0,80
gi|1131324850	putative xaa-pro aminopeptidase [Nyssomyia neivai]	0,80
gi|1131321535	putative nuclear pore complex component sc seh1 [Nyssomyia neivai]	0,79
gi|1131317917	putative eukaryotic translation initiation factor 3 subunit b [Nyssomyia neivai]	0,78
gi|1131325164	putative puromycin-sensitive aminopeptidase, partial [Nyssomyia neivai]	0,77
gi|1131320303	putative vesicle coat protein clathrin heavy chain [Nyssomyia neivai]	0,73
gi|1035819131	translation elongation factor 1-alpha, partial [Phlebotomus alexandri]	0,70
gi|1131327199	putative actin-related protein arp2/3 complex subunit arpc2 [Nyssomyia neivai]	0,70
gi|1131322627	putative acyl-coa synthetase, partial [Nyssomyia neivai]	0,67
gi|1131324752	putative glycogen synthase [Nyssomyia neivai]	0,66
gi|1131318546	putative ubiquitin thioesterase otubain-like protein, partial [Nyssomyia neivai]	0,66
gi|1131324658	putative pfkb family carbohydrate kinase [Nyssomyia neivai]	0,66
gi|1131309041	putative peroxisomal long-chain acyl-coa transporter abc superfamily protein [Nyssomyia neivai]	0,65
gi|1131317833	putative eukaryotic translation initiation factor 2a, partial [Nyssomyia neivai]	0,64
gi|1131320371	putative flotillin [Nyssomyia neivai]	0,64
gi|1131313505	putative juvenile hormone-inducible protein [Nyssomyia neivai]	0,64
gi|1131317563	putative peptide chain release factor 1 erf1 [Nyssomyia neivai]	0,62
gi|1131320377	putative clathrin-associated protein medium chain [Nyssomyia neivai]	0,59
gi|1131323673	putative pyruvate carboxylase, partial [Nyssomyia neivai]	0,54
gi|1131315125	putative type ii serine kinase, partial [Nyssomyia neivai]	0,51
gi|1131319419	putative peroxiredoxin posttranslational modification [Nyssomyia neivai]	0,50
gi|1131322599	putative acyl-coa synthetase [Nyssomyia neivai]	0,50
gi|1131319135	putative molecular chaperone grp170/sil1 hsp70 superfamily protein [Nyssomyia neivai]	0,49

gi|1131320411	putative medium subunit of clathrin adaptor complex [Nyssomyia neivai]	0,49
gi|1131324742	putative 14-alpha-glucan branching enzyme/starch branching enzyme ii [Nyssomyia neivai]	0,47
gi|1131305661	putative conserved plasma membrane protein [Nyssomyia neivai]	0,46
gi|1131325190	putative glutamate decarboxylase/sphingosine phosphate lyase [Nyssomyia neivai]	0,44
gi|1131316895	putative maltase glucoamylase, partial [Nyssomyia neivai]	0,42
gi|1131324702	putative 2-oxoglutarate dehydrogenase e1 subunit [Nyssomyia neivai]	0,39
gi|1131322513	putative acyl-coa synthetase, partial [Nyssomyia neivai]	0,37
gi|1131325064	putative glutamate synthase, partial [Nyssomyia neivai]	0,33
gi|1131325218	putative puromycin-sensitive aminopeptidase [Nyssomyia neivai]	0,31
gi|1131319345	protein disulfide-isomerase [Nyssomyia neivai]	0,31
gi|1131325875	putative glucosamine 6-phosphate synthetase [Nyssomyia neivai]	0,30
gi|1131325869	putative udp-glucose 4-epimerase/udp-sulfoquinovose synthase [Nyssomyia neivai]	0,30
gi|1131317471	putative arginyl-trna synthetase [Nyssomyia neivai]	0,27
gi|1131318564	putative e3 ubiquitin-protein ligase huwe1 [Nyssomyia neivai]	0,23
gi|1131319593	putative peptide exporter abc superfamily protein [Nyssomyia neivai]	0,23
gi|1131327149	putative ca2+-binding actin-bundling protein [Nyssomyia neivai]	-0,18
gi|1131319429	putative heat shock protein 90 [Nyssomyia neivai]	-0,19
gi|1131314465	putative g protein [Nyssomyia neivai]	-0,23
gi|1131312407	putative cell division cycle and apoptosis regulator protein 1, partial [Nyssomyia neivai]	-0,24
gi|1131317603	putative 40s ribosomal protein s17 [Nyssomyia neivai]	-0,30
gi|1131327177	putative ca2+-binding actin-bundling protein [Nyssomyia neivai]	-0,32
gi|1131323798	putative ubiquinone oxidoreductase b16.6 subunit/cell death-regulatory protein [Nyssomyia neivai]	-0,36
gi|1131318312	putative 26s proteasome regulatory complex subunit [Nyssomyia neivai]	-0,39
gi|1131313137	putative enzyme that catalyze nonsubstrate specific conversion [Nyssomyia neivai]	-0,40
gi|1131327161	putative calponin [Nyssomyia neivai]	-0,41
gi|1131316909	putative alpha-amylase [Nyssomyia neivai]	-0,41
gi|1131323902	putative cytochrome c [Nyssomyia neivai]	-0,42

gi|1131320425	putative vacuolar sorting protein vps45/stt10 sec1 family [Nyssomyia neivai]	-0,43
gi|1131323760	putative lipoyltransferase [Nyssomyia neivai]	-0,43
gi|1131316933	putative metalloexopeptidase, partial [Nyssomyia neivai]	-0,45
gi|1131320213	putative signal recognition particle subunit srp68, partial [Nyssomyia neivai]	-0,45
gi|1131319623	putative signal peptidase i [Nyssomyia neivai]	-0,45
gi|1131325142	putative carbon-nitrogen hydrolase [Nyssomyia neivai]	-0,45
gi|1131326287	putative cytochrome [Nyssomyia neivai]	-0,46
gi|1131325042	putative cysteine desulfurase nfs1 [Nyssomyia neivai]	-0,47
gi|1131323555	putative cdgsh iron-sulfur domain-containing protein 2 [Nyssomyia neivai]	-0,47
gi|1131318330	putative 26s proteasome regulatory complex atpase rpt6 [Nyssomyia neivai]	-0,47
gi|1131323938	putative mitochondrial f1f0-atp synthase subunit oscp/atp5 [Nyssomyia neivai]	-0,48
gi|1131309506	putative vacuolar h+-atpase v1 sector subunit f [Nyssomyia neivai]	-0,50
gi|1131323876	putative mitochondrial carnitine-acylcarnitine carrier protein [Nyssomyia neivai]	-0,50
gi|1131315847	putative serine carboxypeptidase, partial [Nyssomyia neivai]	-0,53
gi|1131326713	putative glutathione s-transferase-like protein, partial [Nyssomyia neivai]	-0,53
gi|1131322697	putative 3-oxoacyl coa thiolase [Nyssomyia neivai]	-0,53
gi|1131317567	putative translation initiation factor 1a eif-1a [Nyssomyia neivai]	-0,55
gi|1131311374	putative rna recognition motif found in sra stem-loop-interacting rna-binding protein, partial [Nyssomyia neivai]	-0,55
gi|172045599	RL17_PHLPP RecName: Full=60S ribosomal protein L17	-0,57
gi|1131320531	hypothetical protein [Nyssomyia neivai]	-0,61
gi|1131325036	putative isovaleryl-coa dehydrogenase [Nyssomyia neivai]	-0,63
gi|1131310860	putative rna-binding protein musashi/mrna cleavage and polyadenylation factor i complex, partial [Nyssomyia neivai]	-0,65
gi|1131318601	putative ubiquitin specific peptidase 14 [Nyssomyia neivai]	-0,65
gi|1131318368	putative 26s proteasome regulatory complex atpase rpt6 [Nyssomyia neivai]	-0,71
gi|1131304547	hypothetical protein, partial [Nyssomyia neivai]	-0,72
gi|1131326451	putative glutathione s-transferase 1 isoform d [Nyssomyia neivai]	-0,74
gi|1131317565	putative translation initiation factor 5a eif-5a [Nyssomyia neivai]	-0,76

gi|1131327371	putative microtubule associated complex, partial [Nyssomyia neivai]	-0,76
gi|1131317877	putative 60s acidic ribosomal protein [Nyssomyia neivai]	-0,77
gi|1131317635	putative translation initiation factor 3 subunit g eif-3g, partial [Nyssomyia neivai]	-0,83
gi|1131322729	putative fatty acid-binding protein fabp [Nyssomyia neivai]	-0,83
gi|1131318296	putative 20s proteasome regulatory subunit beta type psmb2/pre1 [Nyssomyia neivai]	-0,84
gi|1131325026	putative cystathionine beta-lyases/cystathionine gamma-synthase, partial [Nyssomyia neivai]	-0,85
gi|1131308238	putative eukaryotic protein of unknown function [Nyssomyia neivai]	-0,88
gi|1131327341	putative actin binding protein [Nyssomyia neivai]	-0,90
gi|1131322925	putative pyridoxal-phosphate dependent enzyme [Nyssomyia neivai]	-0,94
gi|1131322763	putative transthyretin [Nyssomyia neivai]	-1,03
gi|1131320797	putative histone 2a [Nyssomyia neivai]	-1,05
gi|1131320261	putative nuclear transport factor 2 [Nyssomyia neivai]	-1,08
gi|1131326197	putative cytochrome, partial [Nyssomyia neivai]	-1,12
gi|1131310196	putative rna-binding protein musashi/mrna cleavage and polyadenylation factor i complex, partial [Nyssomyia neivai]	-1,17
gi|1131323577	putative cytochrome c1 [Nyssomyia neivai]	-1,45
gi|1131303979	hypothetical protein, partial [Nyssomyia neivai]	-1,46
gi|1131318071	putative ubiquitin-conjugating enzyme e2-17 kda [Nyssomyia neivai]	-1,50
gi|1131317287	putative 60s ribosomal protein l22, partial [Nyssomyia neivai]	-1,54
gi|1131326617	putative dehydrogenase, partial [Nyssomyia neivai]	-1,61
gi|1131306934	protein aael aael012860 aedes aegypti, partial [Nyssomyia neivai]	-1,73
gi|1131326911	putative myofilin [Nyssomyia neivai]	-1,74
gi|1131320793	putative histone 2a, partial [Nyssomyia neivai]	-2,16
gi|1131317879	putative 60s acidic ribosomal protein [Nyssomyia neivai]	-2,36
