# Supplementary material for: Comparative Gut Proteome of Nyssomyia umbratilis from Leishmaniasis Endemic and Non-Endemic Areas of Amazon Reveals Differences in Microbiota and Proteins Related to Immunity and Gut Function
Source: Microorganisms. 2025 Jun 4;13(6):1304. doi: 10.3390/microorganisms13061304 (PMC12195068; doi:10.3390/microorganisms13061304)
Supplement: Supplementary file 1 [file microorganisms-13-01304-s001.zip › Table S2A.pdf]

CN	CC	NPC	PN	PI	PD
1	Red	11	AhcyL1	7227.FBpp0072886	Adenosylhomocysteinase like 1 (AhcyL1) encodes a protein that, together with the product of AhcyL2, belongs to AHCY-like proteins. AhcyL1 and AhcyL2 products have lost their canonical enzymatic functions due to critical mutations in their AHCY-domains. However, via hetero-multimerization, the products of AhcyL1 and AhcyL2 can suppress the activity of the enzyme encoded by Ahcy, which catalyzes the hydrolysis of S-Adenosylhomocysteine. The presence of a N-terminal IRBIT domain confers AhcyL1 and AhcyL2 proteins new functions in Ca [2+] signaling, intracellular pH regulation and productio [...]
1	Red	11	CG7414	7227.FBpp0078095	Eukaryotic translation initiation factor 2A; Functions in the early steps of protein synthesis of a small number of specific mRNAs. Acts by directing the binding of methionyl- tRNAi to 40S ribosomal subunits. In contrast to the eIF-2 complex, it binds methionyl-tRNAi to 40S subunits in a codon-dependent manner, whereas the eIF-2 complex binds methionyl-tRNAi to 40S subunits in a GTP-dependent manner.
1	Red	11	CG7483	7227.FBpp0081324	Eukaryotic initiation factor 4A-III; ATP-dependent RNA helicase. Core component of the splicing-dependent multiprotein exon junction complex (EJC) deposited at splice junctions on mRNAs. Involved in exon definition of genes containing long introns, including the rolled/MAPK gene. Has a role in oskar mRNA localization at the posterior pole of the developing oocyte.
1	Red	11	Dbp80	7227.FBpp0304166	DEAD-box helicase Dbp80; Dead box protein 80 (Dbp80) encodes a putative ATP-dependent RNA helicase (DEAD box helicase), based on sequence similarity to other Dead box proteins, which are involved in mRNA export from the nucleus.
1	Red	11	eEF1alpha1	7227.FBpp0304260	Elongation factor 1-alpha 1; This protein promotes the GTP-dependent binding of aminoacyl- tRNA to the A-site of ribosomes during protein biosynthesis; Belongs to the TRAFAC class translation factor GTPase superfamily. Classic translation factor GTPase family. EF-Tu/EF-1A subfamily.
1	Red	11	eIF3b	7227.FBpp0086098	Eukaryotic translation initiation factor 3 subunit B; RNA-binding component of the eukaryotic translation initiation factor 3 (eIF-3) complex, which is

					involved in protein synthesis of a specialized repertoire of mRNAs and, together with other initiation factors, stimulates binding of mRNA and methionyl-tRNAi to the 40S ribosome. The eIF-3 complex specifically targets and initiates translation of a subset of mRNAs involved in cell proliferation.
1	Red	11	eIF3i	7227.FBpp0078689	Eukaryotic translation initiation factor 3 subunit I; Component of the eukaryotic translation initiation factor 3 (eIF-3) complex, which is involved in protein synthesis of a specialized repertoire of mRNAs and, together with other initiation factors, stimulates binding of mRNA and methionyl-tRNAi to the 40S ribosome. The eIF-3 complex specifically targets and initiates translation of a subset of mRNAs involved in cell proliferation.
1	Red	11	eIF4A	7227.FBpp0297911	Eukaryotic initiation factor 4A; ATP-dependent RNA helicase which is a subunit of the eIF4F complex involved in cap recognition and is required for mRNA binding to ribosome. In the current model of translation initiation, eIF4A unwinds RNA secondary structures in the 5'-UTR of mRNAs which is necessary to allow efficient binding of the small ribosomal subunit, and subsequent scanning for the initiator codon. Involved in germ cell formation.
1	Red	11	eRF3	7227.FBpp0305302	Eukaryotic translation release factor 3 (eRF3) encodes a GTP-binding protein important in the termination of protein translation.
1	Red	11	me31B	7227.FBpp0079565	ATP-dependent RNA helicase me31b; ATP-dependent RNA helicase which is a core component of a variety of ribonucleoprotein complexes (RNPs) that play critical roles in translational repression and mRNA decapping during embryogenesis, oogenesis, neurogenesis and neurotransmission. Recruits core components and translational repressors to some RNP complexes, and mediates RNP aggregation into processing granules such as P-bodies. As part of a RNP complex containing tral, eIF4E1, cup, and pAbp, involved in RNP-mediated translational repression of maternal mRNAs during oogenesis and embryogene [...]
1	Red	11	mt:ND1	7227.FBpp0390631	NADH-ubiquinone oxidoreductase chain 1; Core subunit of the mitochondrial membrane respiratory chain NADH dehydrogenase (Complex I) that is believed to belong to the minimal assembly required for catalysis. Complex I functions in the transfer of electrons from NADH to the respiratory chain. The

					immediate electron acceptor for the enzyme is believed to be ubiquinone (By similarity).
2	Brown	5	ApepP	7227.FBpp0293470	Xaa-Pro aminopeptidase ApepP; Catalyzes the removal of a penultimate prolyl residue from the N-termini of peptides, such as Arg-Pro-Pro. Belongs to the peptidase M24B family.
2	Brown	5	CG5355	7227.FBpp0079637	LP07359p; Serine-type endopeptidase activity; endopeptidase activity; serine-type exopeptidase activity; oligopeptidase activity; carboxylic ester hydrolase activity. It is involved in the biological process described with: proteolysis.
2	Brown	5	Dip-C	7227.FBpp0311237	Dipeptidase C, isoform A; Peptidase activity; aminopeptidase activity; manganese ion binding; dipeptidase activity. It is involved in the biological process described with: proteolysis.
2	Brown	5	Ide	7227.FBpp0271781	Insulin-degrading enzyme; Insulin degrading metalloproteinase (Ide) encodes a metalloprotease that controls growth in a cell-autonomous manner by regulating the level of the insulin-like peptide encoded by Ilp2.
2	Brown	5	TppII	7227.FBpp0086887	Tripeptidyl-peptidase 2; Component of the proteolytic cascade acting downstream of the 26S proteasome in the ubiquitin-proteasome pathway. May be able to complement the 26S proteasome function to some extent under conditions in which the latter is inhibited (By similarity). Efficiently cleaves Ala-Ala-Ala-polypeptide and Pro-Pro-Ala-polypeptide, Val-Leu-Lys-polypeptide only at high concentration. Does not cleave Ala-Phe-Pro-polypeptide nor Pro-Leu-Gly-polypeptide; Belongs to the peptidase S8 family.
3	Dark Golden Rod	4	Acsl	7227.FBpp0305216	Acyl-CoA synthetase long-chain, isoform J; Long-chain fatty acid-CoA ligase activity; palmitoyl-CoA ligase activity.
3	Dark Golden Rod	4	Ldsdh1	7227.FBpp0071071	Lipid droplet subset dehydrogenase 1; Oxidoreductase activity, acting on the CH-OH group of donors, NAD or NADP as acceptor; retinol dehydrogenase activity; Belongs to the short-chain dehydrogenases/reductases (SDR) family.
3	Dark Golden Rod	4	bgm	7227.FBpp0080167	Very long-chain-fatty-acid--CoA ligase bubblegum; Mediates activation of long-chain fatty acids for both synthesis of cellular lipids, and degradation via beta-oxidation. Probably by regulating lipid storage and catabolism, plays a role in neuronal function.

3	Dark Golden Rod	4	pic	7227.FBpp0082177	Piccolo (pic) encodes the Ddb1 protein, which functions to recruit substrate receptors to Cul4-based E3 ubiquitin ligase complexes, which catalyze the ubiquitylation and subsequent destruction of proteins that function in cell growth and proliferation as well as transcription, replication and repair of the genome.
4	Yellow	4	ACC	7227.FBpp0087947	Acetyl-CoA carboxylase (ACC) encodes a ubiquitous metabolic enzyme. It catalyzes the carboxylation of acetyl-CoA to malonyl-CoA, the rate-limiting substrate for fatty acid synthesis. It is essential in the embryo and in the oenocytes (specialized abdominal cells) for the watertightness of the respiratory system. It is required in the fat body for triglyceride storage and in the muscles for locomotor activity.
4	Yellow	4	Arpc2	7227.FBpp0312086	Actin-related protein 2/3 complex subunit 2; Functions as actin-binding component of the Arp2/3 complex which is involved in regulation of actin polymerization and together with an activating nucleation-promoting factor (NPF) mediates the formation of branched actin networks. Seems to contact the mother actin filament (By similarity).
4	Yellow	4	Mdr50	7227.FBpp0086666	Multi drug resistance 50; Efflux transmembrane transporter activity; ATPase-coupled xenobiotic transmembrane transporter activity; ATPase activity; ATPase-coupled transmembrane transporter activity; ATP binding. It is involved in the biological process described with: response to insecticide; response to toxic substance; xenobiotic transport; transmembrane transport; renal tubular secretion.
4	Yellow	4	Vps13	7227.FBpp0301727	Vacuolar protein sorting-associated protein 13; Plays a role in the survival of neurons by maintaining protein homeostasis in the central nervous system. May function as part of a lysosomal degradation pathway.
5	Olive	4	EMC1	7227.FBpp0308770	ER Membrane protein Complex 1 (EMC1) encodes a subunit of the ER membrane protein complex (EMC). In fly retinas, EMC has been shown to be essential for the biosynthesis of multi-pass membrane proteins, but not for single-pass membrane proteins and secretory proteins.

5	Olive	4	Ostgamma	7227.FBpp0079233	Oligosaccharide transferase gamma subunit (Ostgamma) encodes a subunit of the oligosaccharyltransferase complex involved in cell migration and cellular encapsulation response.
5	Olive	4	Stt3A	7227.FBpp0311838	Catalytic subunit 3A of the oligosaccharyltransferase complex, isoform A; Dolichyl-diphosphooligosaccharide-protein glycotransferase activity. It is involved in the biological process described with: protein N-linked glycosylation via asparagine; post-translational protein modification.
5	Olive	4	Stt3B	7227.FBpp0084161	Catalytic subunit 3B of the oligosaccharyltransferase complex; Dolichyl-diphosphooligosaccharide-protein glycotransferase activity. It is involved in the biological process described with: protein N-linked glycosylation via asparagine; post-translational protein modification.
6	Light Green	3	CG15117	7227.FBpp0085778	Beta-glucuronidase; Plays an important role in the degradation of dermatan and keratan sulfates; Belongs to the glycosyl hydrolase 2 family.
6	Light Green	3	Psa	7227.FBpp0072583	Puromycin sensitive aminopeptidase, isoform C; Peptide binding; zinc ion binding; metalloaminopeptidase activity. It is involved in the biological process described with: peptide catabolic process; proteolysis.
6	Light Green	3	beta-Man	7227.FBpp0078603	Beta-Mannosidase, isoform A; Beta-mannosidase activity. It is involved in the biological process described with: glycoprotein catabolic process.
7	Green 2	3	Flo1	7227.FBpp0086485	Flotillin-1; May act as a scaffolding protein within caveolar membranes, functionally participating in formation of caveolae or caveolae-like vesicles.
7	Green 2	3	Hmt-1	7227.FBpp0082642	Heavy metal tolerance factor 1; It is involved in the biological process described with: heme transport; transmembrane transport; response to cadmium ion.
7	Green 2	3	Pmp70	7227.FBpp0307156	Peroxisomal membrane protein 70 kDa, isoform A; Protein homodimerization activity; ATPase-coupled transmembrane transporter activity; ATP binding; ATPase activity. It is involved in the biological process described with: long-chain fatty acid import into peroxisome.
8	Green	3	AP-2mu	7227.FBpp0083646	Adaptor Protein complex 2, mu subunit (AP-2mu) encodes a component of the AP-2 adaptor complex, which recruits certain transmembrane proteins into clathrin-coated pits for endocytic internalization.

8	Green	3	crq	7227.FBpp0077747	Croquemort (crq) encodes a member of the scavenger receptor class B sub-family. It is expressed in plasmatocyte/macrophages and promotes apoptotic cell clearance. In epidermal cells it plays a role in phagosome maturation in both dendrite pruning and injury.
8	Green	3	rdog	7227.FBpp0084756	Red dog mine, isoform A; ATP binding; ATPase-coupled transmembrane transporter activity; ATPase activity. It is involved in the biological process described with: transmembrane transport.
9	Blue	3	Kr-h2	7227.FBpp0078823	Krueppel homolog 2; Member of the dosage-dependent hierarchy effective upon white gene expression; Belongs to the PER33/POM33 family.
9	Blue	3	Sec13	7227.FBpp0311988	Protein SEC13 homolog; Functions as a component of the nuclear pore complex (NPC) and the COPII coat (By similarity). At the endoplasmic reticulum, SEC13 is involved in the biogenesis of COPII-coated vesicles (By similarity). Recruited to transcriptionally active chromatin at the time of transcription initiation by RNA polymerase II. Required for proper expression of ecdysone-responsive genes such as Eip74EF and Eip75B during larval development. Required for reactivation of transcription after heat shock. Required for nuclear import of phosphorylated Mad via importin msk. Has no role i [...]
9	Blue	3	Yip1d1	7227.FBpp0080011	Protein YIPF; It is involved in the biological process described with: positive regulation of synapse pruning; spermatogenesis.
10	Light Sky Blue	2	Sptr	7227.FBpp0071184	Sepiapterin reductase (Sptr) encodes a sepiapterin reductase involved in the biosynthesis of tetrahydrobiopterin.
10	Light Sky Blue	2	for	7227.FBpp0088350	cGMP-dependent protein kinase, isozyme 2 forms cD4/T1/T3A/T3B; Foraging (for) encodes a serine/threonine kinase and a member of the protein kinase G family. For roles include feeding, locomotion, metabolism, development, olfactory habituation, learning and memory, stress, social behavior, as well as Malpighian tubule, heart, muscle and synaptic function.
11	Orchid 2	2	alphaCOP	7227.FBpp0072694	Coatomer subunit alpha; The coatomer is a cytosolic protein complex that binds to dilysine motifs and reversibly associates with Golgi non-clathrin-coated vesicles, which further mediate biosynthetic protein transport from the ER, via the Golgi up to the trans Golgi network.

11	Orchid 2	2	beta'COP	7227.FBpp0080048	Coatomer subunit beta; The coatomer is a cytosolic protein complex that binds to dilysine motifs and reversibly associates with Golgi non-clathrin- coated vesicles, which further mediate biosynthetic protein transport from the ER, via the Golgi up to the trans Golgi network. Coatomer complex is required for budding from Golgi membranes, and is essential for the retrograde Golgi-to-ER transport of dilysine-tagged proteins (By similarity).
12	Purple	2	Nha1	7227.FBpp0079002	Na[+]/H[+] hydrogen antiporter 1 (Nha1) encodes a chloride transmembrane transporter belonging to the CPA2 family of cation/proton antiporter family.
12	Purple	2	kcc	7227.FBpp0309966	Kazachoc, isoform G; Kazachoc (kcc) encodes a potassium:chloride symporter that contributes to seizure susceptibility.
13	Orchid	2	Plap	7227.FBpp0310466	Phospholipase A2 activator protein, isoform A; Ubiquitin binding. It is involved in the biological process described with: proteasome-mediated ubiquitin-dependent protein catabolic process; ubiquitin recycling.
13	Orchid	2	Rpn7	7227.FBpp0083687	26S proteasome non-ATPase regulatory subunit 6; Acts as a regulatory subunit of the 26S proteasome which is involved in the ATP-dependent degradation of ubiquitinated proteins.
14	Pink	1	Chc	7227.FBpp0089398	Clathrin heavy chain (Chc) encodes a protein that forms part of the clathrin complex, which is the major component of coated vesicles.

Supplementary table S2A- String MCL Cluster MAN- CN- Cluster number; CC- Cluster color; NPC- Number of proteins in the cluster; PN- protein name; PI- Protein identification; PD- Protein description.
